# Utilisation of rehabilitation services for non-migrant and migrant groups of higher working age in Germany – results of the lidA cohort study

**DOI:** 10.1186/s12913-019-4845-z

**Published:** 2020-01-10

**Authors:** Chloé Charlotte Schröder, Maria Dyck, Jürgen Breckenkamp, Hans Martin Hasselhorn, Jean-Baptist du Prel

**Affiliations:** 1 Department of Occupational Health Science, University of Wuppertal, Gaußstraße 20, D-42097 Wuppertal, Germany; 2 Office Dep. 3 - Epidemiology & International Public Health, School of Public Health, Bielefeld University, Universitätsstraße 25, D-33615, Bielefeld, Germany; 3 Health Services Research Unit, Faculty of Health, Witten/Herdecke University, Alfred-Herrhausen-Straße 50, D-58448 Witten, Germany

**Keywords:** utilisation, rehabilitation, migrants, retirement, employee participation, cohort study

## Abstract

**Background:**

An ageing and a shrinking labour force implies that the prevention of a premature exit from work due to poor health will become more relevant in the future. Medical rehabilitation is a health service that aims at active participation in working life. The provision of this service will be relevant for an increasing part of the ageing labour force, namely, employees with a migrant background and their different subgroups. Thus, this study examines whether first- and second-generation employees with migrant background differ from non-migrants in their utilisation of rehabilitation services and whether within the subsample of migrant employees, those persons with foreign nationality differ from those with German nationality.

**Methods:**

Socially insured employees born in 1959 or 1965 were surveyed nationwide in 2011 as part of the lidA cohort study (*n*=6303). Survey data of the first study wave were used to identify the dependent variable of the utilisation of rehabilitation (in- and outpatient), the independent variable of migrant status and the covariates of sociodemographic, work- and non-work-related factors. Applying bivariate statistics with tests of independence and block-wise logistic regressions, differences between the groups were investigated. Additionally, average marginal effects were computed to directly compare the adjusted models.

**Results:**

The study showed that first-generation migrants had a significantly lower likelihood of utilising outpatient rehabilitation than non-migrants (fully adj. OR 0.42, 95% CI 0.22-0.82) and that average marginal effects indicated higher differences in the full model than in the null model. No significant differences were found between the first- or second-generation migrants and non-migrants when comparing the utilisation of inpatient rehabilitation or any rehabilitation or when analysing German and foreign employees with migrant background (*n*=1148).

**Conclusions:**

Significant differences in the utilisation of outpatient rehabilitation between first-generation migrants and non-migrants were found, which could not be explained by sociodemographic, work- and non-work-related factors. Thus, further factors might play a role. The second-generation migrants resemble the non-migrants rather than their parent generation (first-generation migrants). This detailed investigation shows the heterogeneity in the utilisation of health services such as medical rehabilitation, which is why service sensitive to diversity should be considered.

## Introduction

Demographic change affects many domains in industrialised countries, including the ageing and shrinking labour force. In Germany, as a countermeasure, the statutory retirement age was raised leading to prolonged working lives and a higher proportion of older employees [1]. Along with the ageing of the labour force, the risk of poor health and functioning elevates with increasing age, which often leads to a premature exit from working life and rising costs for social security systems [1–3].

An ageing labour force and an increasing number of employees with functional limitations imply that the prevention of premature exit from work due to poor health will become increasingly relevant in the future. Therefore, prevention, rehabilitation and reintegration will gain relevance in working life, especially medical rehabilitation aiming at continued work participation [4]. When the ability to work is at risk or if it is impaired due to poor health or functioning, rehabilitation can improve or restore work ability or inhibit its deterioration to prevent premature work exits [4–6]. In Germany, the system of rehabilitation is quite unique. The legal foundation is set by the social security system in Germany, where five statutory branches work independently from one another. These are the statutory health, pension, accident, unemployment and nursing care insurance. The membership for all employees (except civil servants and employees over a certain income threshold) is compulsory. The exempted people can decide whether they want to be statutorily or privately insured. Therefore, depending on the situation of the concerned person, different rehabilitation providers can be responsible, e.g., the pension, the accident or the health insurance. Briefly, the pension insurance takes over the costs when the person is employed, the accident insurance takes over when rehabilitation is needed because of an occupational accident and the health insurance takes over in most other cases. To obtain access, the person himself or herself has to apply for rehabilitation with the recommendation of a physician. As part of the rehabilitation, different interventions can be used, such as medical rehabilitation, which takes place in rehabilitation clinics, or occupational rehabilitation, which includes interventions at the workplace, or social rehabilitation, which includes several assistance services, e.g., those for mobility [4, 6, 7]. This study mainly focuses on medical rehabilitation. Overall, each year, approximately one million medical rehabilitation services are approved by the main provider, the pension insurance, mostly for musculoskeletal disorders, cancer or mental disorders. These programmes are (mostly) provided on an inpatient as well as outpatient basis, lasting on average 22 to 24 days or 28 days for mental disorders [4, 6].

In this context, it is important to note that the older labour force in Germany is heterogeneous. For example, the proportion of employees with a migrant background (EMB) is continuously growing, e.g., from 16.2% in 2010 to 23.9% in 2018 [8, 9]. The largest proportion of persons with a migrant background (PMB) in Germany are resettlers from Eastern Europe and the former Soviet Union, as well as persons of Turkish and Polish origin [9, 10]. Therefore, PMB constitute a heterogeneous group with regard to their origin, culture, religion and education [11, 12]. Concerning health, only certain health outcomes with different definitions of migrant background have been researched so far, so that further studies are required. According to the existing literature, it is not conclusive that PMB have poorer health in general than non-PMB, and there is a need to differentiate between subgroups and outcomes. Another limitation of previous studies is the lack of sociodemographic data on PMB, which often account for health status compared to non-PMB [13].

PMB comprise persons born outside of Germany (first-generation, G1) and persons born in Germany, but with one or both parents born abroad (second-generation, G2) [9, 10, 14]. PMB can either be German or foreign nationals, depending on their place of birth, making the criterion ‘nationality’ less suitable for identifying this group. When focusing on older employees, it must be considered that in Germany many PMB will soon reach the statutory retirement age themselves as 37.3% of them were over 45 years old in 2018 [9].

It is known that EMB, especially those with foreign nationality, more frequently suffer from occupational accidents and diseases and that they retire earlier with a disability pension compared to employees with German nationality [15, 16]. This difference could be attributed not only to poor health due to more physically demanding occupations and further social inequalities that this group experiences but also to lower utilisation of health services [13, 15, 17, 18]. Medical rehabilitation constitutes one of these health services that aims at active participation in working life. In Germany, persons with migrant background, especially those with a foreign nationality, are less likely to utilise rehabilitation services than non-migrants [19–21]. This is possibly due to barriers such as lack of information, language problems, illiteracy or cultural barriers [22–24].

However, current studies on migrants’ utilisation of rehabilitation services in Germany have several limitations. Quantitative studies are often based on secondary data, such as process data from rehabilitation providers (e.g., pension insurance). In most such data sets, the migrant background is solely indicated by ‘nationality”, thus not permitting a differentiation in migrant backgrounds and misclassifying a large proportion of people, up to 48% (9.4 million foreign nationals out of 19.3 million persons with a migrant background) [9]. Furthermore, the findings of the qualitative studies are not representative. Experts in the field have consequently identified a need for large-scale primary studies on migrants’ utilisation of rehabilitation services in Germany [25].

To our knowledge, representative studies in Germany investigating the utilisation of in- and outpatient rehabilitative care in older employees with distinct differentiation between migrant backgrounds are missing. Additionally, there are no investigations as yet that would compare groups within PMB or EMB to identify possible contrasting behaviours such as first- and second-generation behaviours or behaviours related to nationality. Obtaining German nationality is accompanied by considerable simplifications in one’s life and a higher willingness to integrate into German society [17], which may have a potential influence on the utilisation of rehabilitation. Thus, the consideration of the heterogeneity in persons with migrant background is essential as subgroups might act differently in the utilisation of health services and in terms of medical rehabilitation.

Therefore, the current study primarily investigates, whether first- and second-generation employees with migrant background differ from employees without migrant background in their utilisation of rehabilitation services. Second, the study investigates the subsample of migrant employees with foreign nationality as to whether they differ from those migrant employees with German nationality in their utilisation of these services. Moreover, the impact of different sociodemographic, work- and non-work-related factors is investigated to explain group differences.

## Methods

### Study design and participants

The lidA (leben in der Arbeit) cohort study examines the work, age, health and work participation of an ageing workforce in Germany. Two birth cohorts (1959 and 1965) were chosen as being part of the German ‘baby boomer’ generation, constituting the older labour force and moving towards retirement with less options for early retirement than earlier retirement cohorts. The age difference between the cohorts was set to investigate possible cohort effects other than age or time (period) effects, which can occur during follow-up in intervals. The lidA-study population was selected in a two stage sampling process from the ‘Integrated Employment Biographies’ (IEB) dataset, which is the data register from the German Federal Employment Agency. Within sampling, in the first stage, an area selection of 222 sample points was carried out; the points were drawn proportionally to the population and spread across the entire Federal Republic of Germany. The second selection stage consisted of the selection of employees subject to social security contributions at each sample point. The dataset therefore contains all socially insured employees born in 1959 or 1965 in Germany who were employed on the reference date of 31 December 2009, which covers 80% of the German working population. The participants were interviewed at home for each assessment wave, based on computer assisted personal interviews (CAPI) covering topics such as work, health and private life [26, 27]. To date, three waves of assessment have been performed in 2011, 2014 and 2018. The lidA study was approved by the Ethics Committee of the University of Wuppertal (dated from 05/12/2008 and 20/11/2017, MS/BB 171025 Hasselhorn). The datasets analysed in the current study are available as a scientific use file at the Research Data Centre of the German Federal Employment Agency at the Institute of Employment Research [https://fdz.iab.de/en/FDZ_Individual_Data/lidA.aspx] [28].

For the present analysis, data from the first study wave in 2011 were used, where 6585 participants took part. At this point in time, the participants were 46 and 52 years old. Participants in full-time, part-time, irregular or marginally employed positions (at least 1h/week) in 2011 were included in the sample (n=6339). Due to the sampling specification, employees such as civil servants, self-employed persons and freelancers were excluded. As all interviews in the lidA study were performed in German, no interviews were realised with persons not able to communicate sufficiently in the German language. Further, 36 participants with undefined migrant status were excluded. As a result, the sample consists of 6303 individuals.

### Measurements

#### The outcome of rehabilitation services

The primary outcome was ‘utilised medical rehabilitation’, which was self-reported with the questionnaire. Participants were asked to report whether they had utilised an in- or outpatient rehabilitation service in the previous three years. All outcomes were generated as a binary variable indicating general, in- or outpatient rehabilitation vs. no utilisation of rehabilitation, respectively.

#### Migrant background

The lidA cohort study allows distinguishing migrant groups by means of specific indicators, as recommended by Schenk et al. [29].

Migrant background was operationalised based on the self-reported country of birth, nationality of the participants and the country of birth of each of their parents. Participants with place of birth in Germany, German nationality and with both parents born in Germany, were the reference group (non-EMB). The first migrant generation (G1 EMB) was defined according to the definition of the German Federal Statistical Office [8, 9] as persons who were born abroad and who had immigrated to Germany, meaning that their country of birth is not Germany. Participants with German citizenship not born in Germany and with both parents born in Germany were included in G1 EMB because of the strictly defined reference group.

The second migrant generation (G2 EMB) was classified as participants born in Germany with at least one parent born abroad. For the second group comparison, the subsample of employees with migrant background (EMB) was split into those employees with German/dual and foreign nationality (German and foreign EMB).

#### Covariates

Sociodemographic, work-related, and non-work-related factors were included as covariates in the analysis to describe group differences and to control potential confounders.

##### Sociodemographic factors

As sociodemographic factors, the year of birth (1959/1965), sex (male/female) and occupational class were considered as covariates. As sex is an important determinant for health service utilisation, we tested for interaction effects between sex and migrant background, but this was neither significant for general, in- or outpatient rehabilitation, nor for sex and nationality in EMB.

Occupational classes as classified by Blossfeld were used, which are based on the German Classification of Occupation of the Federal Employment Agency in the 1988 version [30]. The occupational classes were operationalised from twelve groups into the three of categories highly qualified, qualified, and un-/semi-skilled in consideration of a validation study with data from the micro-census [31]. These groups may also indirectly represent educational qualifications, mostly a precondition for the later occupational class in Germany [32].

##### Work-related factors

Specific physical and psychosocial work exposure variables that are known to be associated with poor health were selected [33, 34]. A range of such variables is considered in checklists recommended by the German pension insurance to assess the need for rehabilitation [35, 36]. These were included in our analyses to determine whether work-related factors could provide an additional explanatory power for the utilisation of rehabilitation services beyond the health aspect.

The following psychosocial work factors were considered: quality of leadership, own influence at work and work-privacy conflict, all based on the Copenhagen Psychosocial Questionnaire (COPSOQ II, middle version, only short version for the variable work-privacy conflict) [37, 38]. Influence at work and quality of leadership were assessed with three items (including five categories each), while work-privacy conflict was measured with two items (with four categories each). Each item was measured categorically and, for analysis, each was transformed to a value range from 0 (minimum value, i.e., never ever) to 100 (maximum value, i.e., always). All three scales were built by the mean value of the single items included in each scale. The cut-off value for the dichotomisation in the categories low and high was set at 50 for influence at work and quality of leadership and at 67 for work-privacy conflict [37–39].

Work-related stress, another psychosocial work factor, was assessed and analysed with the long version of the effort-reward imbalance (ERI) questionnaire by Siegrist et al. [40, 41], which was implemented in the lidA questionnaire. Imbalance was measured with the ERI ratio formed as the quotient of the effort and the reward scales including a weighting factor for the different numbers of items in the nominator and denominator. The ERI ratio was calculated from the 17 items and could be used as a continuous measure or transformed into tertiles representing low, medium or high work stress. For bivariate statistics, the median and interquartile range were used to compare the groups with different migrant background, for further multiple analyses of the tertiles. Values close to zero express the preferable situation with low work stress while values above 1.0 indicate a very high ERI imbalance, meaning higher personal work stress [40, 41].

Occupational physical load was measured with two variables. First, the physical environmental factors, meaning the combination of variables comprising exposure to cold, heat, humidity and noise, and second, physical burdensome factors, such as working while leaning over, working on the knees, working one-sided or doing heavy lifting and carrying [42]. Participants were supposed to indicate with a graded answer scheme how much of the working time they are exposed to such work. Participants were classified as being exposed if they – in either variable – indicated exposure as more than half of their working time. This cut-off was chosen in accordance with the SF12 single item (see below), as people working more than half of their working time had increased poor health.

##### Non-work-related factors

Self-rated health in general was parametrised by the single item Short Form-12 Health Survey (SF-12) [43], containing the following question: ‘In general, would you say your health is…’, with a 5-category Likert response scale of very good, good, satisfactory, poor or very poor. The categories satisfactory to very poor were summarised as poor, while the other categories presented good health according to international procedures. Several studies showed that this widely used health indicator is a predictor of later morbidity and mortality [44, 45].

The second non-work-related variable was the main language spoken at home, which was categorised into mostly German and mostly another language. Here, this variable was not used to identify third-generation migrants (the persons themselves and with parents born in Germany but whose mother tongue was not German) but to account for possible differences between these migrant groups.

All mentioned items without any references were self-developed questionnaire items. The English translation of the items can be found in the attachment (see Additional file 1).

### Statistical analysis

Descriptive and bivariate statistics including cross tables, Chi^2^- and Kruskal-Wallis tests were used to characterise the full sample separated for the three groups of migrant background. To investigate whether these groups differed in terms of the utilisation of rehabilitation in the multivariate analysis, block-wise logistic regressions were performed while adjusting for sociodemographic, work-related and non-work-related factors. This was carried out separately for the outcome of general, inpatient and outpatient rehabilitation. Some variables had missing data (MD): the percentage of MD ranged from 0.05% (occupational physical load) to 20.0% (effort-reward imbalance). Up to 1900 cases were lost, depending on the variables included in the regression models. Consequently, missing data were replaced by the fully conditional specification method, a multiple imputation approach, to increase the power of the regression analysis and to reduce bias [41]. Using ten iterations, twenty datasets were created. The imputation model included all variables from the analysis model as introduced before and additional supporting variables on school and occupational education as well as quantitative demands. The imputed datasets were used for the hierarchical logistic regressions.

To answer the second research question, the subsample of employees with migrant background were additionally separated into employees with German or foreign nationality. Subsequently, descriptive and bivariate analyses were performed to compare these two groups (incl. Chi^2^- and Wilcoxon-Mann-Whitney-test), as well as block-wise logistic regression to investigate differences between these two groups with respect to the utilisation of general rehabilitation. Separated analyses for in- and outpatient rehabilitation were not possible due to the small number of events (utilisation of rehabilitation) in German and foreign EMB.

Additionally, for all logistic regressions, average marginal effects (AMEs) were computed with SAS 9.4.

AMEs allow us to compare the results of nested models that are otherwise possibly biased by unobserved heterogeneity. The latter represents influences on the dependent variable by unobserved or unconsidered variables that can cause false interpretation in e.g. logistic regression as odds ratios also demonstrate unobserved heterogeneity. Therefore, the interpretation of the regression coefficient in models with a non-linear transformation (e.g., logit in logistic regression) is typically not as straightforwardly interpretable as in ordinary least-squares regression. The coefficient represents the influence of each variable on the linear scale of the outcome, not the probability scale of the observed outcome. AMEs are based on derivatives of the logistic probability distribution functions, which measure the average conditional effects. The AME shows for each variable in a regression model how much the event probability changes when the independent variable increases by one unit or rather when a binary independent variable changes its level [46, 47].

In all statistical tests, p-values (two-tailed) < 0.05 were considered statistically significant. For the logistic regressions, Nagelkerke’s Pseudo-R^2^ is presented as a measure for comparing competing models. All statistical analyses (except the average marginal effects) were performed using SPSS version 25.0 (IBM Corp., Armonk, NY).

## Results

### Descriptive and bivariate analysis

The baseline characteristics of the 6303 participants included in the analysis are given in Table 1. A total of 12.8% (n=808) of the participants had used any type of rehabilitation (primary outcome) in the last three years. These were mainly inpatient services rather than outpatient services. No significant differences in utilisation were found between the three groups of non-EMB, G1 EMB and G2 EMB. However, a comparatively low proportion of outpatient rehabilitation (2.3%) among G1 EMB was observed. Significant differences were found for covariates, e.g., occupational class, where G1 EMB exhibited considerably lower occupational levels than the other two groups. Additionally, in comparison, G1 EMB significantly more often reported low influence at work (62.4%), were more often exposed to physical work exposures (39.0 and 37.7%), reported poor health more frequently (50.1%) and fairly more frequently spoke a language other than German at home (36.4%) than the other groups investigated.

**Table 1:** Characteristics of the study sample of socially insured employees, as specified by migrant background (n=6303)

	Non-EMB (*n*=5153)	G1 EMB (*n*=699)	G2 EMB (*n*= 451)	*p*-value^a^
Utilisation of rehabilitation services [n (%)], m=3				
None	4485 (87.1)	617 (88.3)	390 (86.5)	0.612^b^
Yes	665 (12.9)	82 (11.7)	61 (13.5)	
Inpatient	440 (8.5)	66 (9.4)	43 (9.5)	0.109^c^
Outpatient	225 (4.4)	16 (2.3)	19 (4.0)	
Year of birth 1959 [n (%)]	2291 (44.5)	292 (41.8)	188 (41.7)	0.244
Female sex [n (%)]	2743 (53.2)	345 (49.4)	255 (56.5)	0.047*
Occupational class [n (%)], m=63				
Highly qualified	1001 (19.6)	77 (11.2)	89 (19.9)	
Qualified	2238 (43.9)	189 (27.4)	194 (43.3)	< 0.001***
Un-/semi-skilled	1863 (36.5)	424 (61.4)	165 (36.8)	
Low quality of leadership [n (%)], m=472	1479 (31.0)	194 (30.4)	139 (33.3)	0.558
High work-privacy conflict [n (%)], m=63	1182 (23.1)	155 (22.6)	103 (23.1)	0.944
Low influence at work [n (%)], m=1058	2430 (55.7)	313 (62.4)	202 (53.0)	0.008**
Work stress, ERI [Mdn (IQR)], m=1238	0.45 (0.25)	0.44 (0.24)	0.46 (0.24)	0.758^d^
Exposed to physical environmental factors [n (%)], m=3	1417 (27.5)	272 (39.0)	137 (30.4)	< 0.001***
Exposed to physical burdensome factors [n (%)], m=3	1613 (31.3)	263 (37.7)	139 (30.8)	0.003**
Poor self-rated health [n (%)]	2292 (44.5)	350 (50.1)	213 (47.2)	0.014*
Home language mostly German [n (%)]	5147 (99.9)	445 (63.6)	448 (99.4)	< 0.001***

*m* number of missing values due to respondents not responding to the item, *Mdn* median, *IQR* interquartile range; * *p* < 0.05, ** *p* < 0.01, *** *p* < 0.001

^a^tested with Chi^2^-test if not otherwise specified

^b^testing dichotomous variable of utilisation of rehabilitation (yes/no)

^c^testing trichotomous variable of utilisation of rehabilitation (no/inpatient/outpatient)

^d^tested with Kruskal-Wallis test

### Association between utilisation of either general, outpatient or inpatient rehabilitation and migrant background in 2011

Comparing the general utilisation of rehabilitation services in the logistic regression model, G1 EMB had a somewhat lower and G2 EMB had a slightly higher odds of utilisation than non-EMB, when considering all explanatory variables (G1 EMB: OR 0.91, 95% CI 0.68-1.23; G2 EMB: OR 1.05, 95% CI 0.79-1.39). Nevertheless, utilisation did not differ significantly from that among non-EMB, neither for G1 nor for G2 EMB (see Table 2). Further adjusting the models with sociodemographic and work-related variables first decreased the probability of the utilisation of rehabilitation (see AMEs) for G1 EMB (to 1.7%-points) and then increased the probability for G2 EMB (to 0.72%-points), while holding the covariates at a constant value. However, in the final model 3 the probabilities declined again.

**Table 2:** Association between utilisation of rehabilitation services (general/ outpatient/ inpatient) and migrant background in 2011

	Model 0	Model 1^a^	Model 2^b^	Model 3^c^	Reduction^d^ (%)
General rehabilitation services (*n*=6303/ n_events_=808)					
OR (95%-CI)					
Non-EMB	Ref.	Ref.	Ref.	Ref.	
G1 EMB	0.90 (0.72-1.11)	0.86 (0.67-1.10)	0.86 (0.67-1.10)	0.91 (0.68-1.23)	-1.11
G2 EMB	1.06 (0.91-1.22)	1.06 (0.92-1.23)	1.07 (0.80-1.41)	1.05 (0.79-1.39)	0.94
AME					
Non-EMB	Ref.	Ref.	Ref.	Ref.	
G1 EMB	-0.0122	-0.0168	-0.0169	-0.0104	14.75
G2 EMB	0.0060	0.0068	0.0072	0.0047	21.67
R^2^	0.000	0.006	0.017	0.057	
Inpatient rehabilitation services (*n*=6044/ n_events_=549)					
OR (95%-CI)					
Non-EMB	Ref.	Ref.	Ref.	Ref.	
G1 EMB	1.09 (0.82-1.45)	1.04 (0.79-1.37)	1.04 (0.78-1.37)	1.16 (0.84-1.60)	-6.42
G2 EMB	1.12 (0.95-1.33)	1.14 (0.84-1.53)	1.14 (0.82-1.58)	1.10 (0.79-1.54)	1.79
AME					
Non-EMB	Ref.	Ref.	Ref.	Ref.	
G1 EMB	0.0071	0.0027	0.0031	0.0118	-66.20
G2 EMB	0.0096	0.0104	0.0104	0.0075	21.88
R^2^	0.000	0.012	0.028	0.078	
Outpatient rehabilitation services (*n*=5754/ n_events_=259)					
OR (95%-CI)					
Non-EMB	Ref.	Ref.	Ref.	Ref.	
G1 EMB	0.52 (0.31-0.85)*	0.51 (0.30-0.85)*	0.50 (0.30-0.84)**	0.42 (0.22-0.82)*	19.23
G2 EMB	0.92 (0.59-1.44)	0.92 (0.56-1.53)	0.93 (0.57-1.53)	0.91 (0.56-1.50	1.09
AME					
Non-EMB	Ref.	Ref.	Ref.	Ref.	
G1 EMB	-0.0238	-0.0292	-0.0302	-0.0382	-60.50
G2 EMB	-0.0035	-0.0033	-0.0028	-0.0037	-5.71
R^2^	0.004	0.006	0.012	0.023	

*OR* Odds Ratio, *CI* confidence interval, *Ref*. reference, *AME* Average marginal effect, *R*^*2*^ Nagelkerke pseudo-R^2^; * *p* < 0.05, ** *p* < 0.01

^a^adjusted for year of birth, sex, and occupational class

^b^further adjusted for the quality of leadership, influence at work, work-privacy conflict, work stress (ERI), and phys. environmental and burdensome factors

^c^further adjusted for self-rated health and language at home

^d^reduction of effect size between model 0 and 3

For the utilisation of inpatient rehabilitation, no significant differences between the migrant groups were observed in the analysis. However, higher odds ratios for the utilisation of inpatient rehabilitation were detected for both EMB groups compared to non-EMB (G1 EMB: fully adj. OR 1.16, 95% CI 0.84-1.60; G2 EMB: fully adj. OR 1.10, 95% CI 0.79-1.54). Average marginal effects showed the highest/lowest probability for inpatient rehabilitation in model 3 while the odds ratios did not indicate a large difference.

Analysing the utilisation of outpatient rehabilitation, G1 EMB had significantly lower odds of receiving outpatient rehabilitation than non-EMB in the null model. When adding all explanatory covariates, the direction of the effect for G1 EMB remained the same (OR 0.42, 95% CI 0.22-0.82). Throughout all models, G2 EMB had somewhat lower odds ratios of utilising outpatient rehabilitation. The average marginal effects showed the lowest probability for inpatient rehabilitation in model 3. The difference in the AMEs between the null and the final model indicated an increase of the effect by 60%.

### Subsample analysis of employees with migrant backgrounds stratified by nationality

The analyses of the second research question were performed by separating EMB into those persons with German and foreign nationality. The results are shown in Tables 3 and 4. In the descriptive and bivariate analysis (Table 3), significant group differences were found for year of birth, sex, occupational class and main language spoken at home. The group of participants with foreign EMB were more often younger (67.9%), male (54.9%), mainly belonging to a lower occupational class (63.9%) and more often speaking another language at home than German EMB (47.6%).

**Table 3:** Characteristics of employees with migrant background, specified by nationality, *n*=1148

	German EMB (*n* = 902)	Foreign EMB (*n* = 246)	*p*-value^a^
Utilisation of out- or inpatient rehabilitation [n (%)]	115 (12.7)	28 (11.4)	0.565
Year of birth 1959 [n (%)]	400 (44.3)	79 (32.1)	< 0.001***
Female sex [n (%)]	488 (54.1)	111 (45.1)	0.012*
Occupational class [n (%)], m=12			
Highly qualified	140 (15.7)	26 (10.7)	< 0.0005***
Qualified	321 (36.0)	62 (25.4)	
Un-/semi-skilled	431 (48.3)	156 (63.9)	
Low quality of leadership [n (%)], m=94	266 (31.9)	65 (29.7)	0.537
High work-privacy conflict [n (%)], m=17	206 (23.2)	52 (21.4)	0.554
Low influence at work [n (%)], m=266	405 (57.6)	109 (60.9)	0.426
Work stress, ERI [Mdn (IQR)], m=301	0.45 (0.25)	0.43 (0.23)	0.260^b^
Exposed to physical environmental factors [n (%)], m=1	327 (36.3)	81 (33.1)	0.355
Exposed to physical burdensome factors [n (%)], m=1	308 (34.1)	92 (37.6)	0.321
Poor self-rated health [n (%)]	449 (49.8)	112 (45.5)	0.237
Home language mostly German [n (%)]	764 (84.7)	129 (52.4)	< 0.0005***

*m* number of missing values due to respondents not responding to the item, *Mdn* median, *IQR* interquartile range; * *p* < 0.05, *** *p* < 0.001

^a^tested with Chi^2^-test if not otherwise specified

^b^tested with Wilcoxon-Mann-Whitney-test

**Table 4:** Association between utilisation of general rehabilitation services and nationality in employees with migrant background

	Model 0	Model 1^a^	Model 2^b^	Model 3^c^	Reduction^d^ (%)
Rehabilitation services in general (*n*=1148/ n_events_=143)					
OR (95%-CI)					
German EMB	Ref.	Ref.	Ref.	Ref.	
Foreign EMB	0.88 (0.57-1.36)	0.87 (0.57-1.35)	0.86 (0.55-1.35)	0.91 (0.57-1.46)	-3.41
AME					
German EMB	Ref.	Ref.	Ref.	Ref.	
Foreign EMB	-0.0141	-0.0147	-0.0166	-0.0105	25.53
R^2^	0.001	0.005	0.018	0.026	

*OR* Odds Ratio, *CI* confidence interval, *Ref*. reference, *AME* Average marginal effect, *R*^*2*^ Nagelkerke pseudo-R^2^

^a^ adjusted for year of birth, sex, and occupational class

^b^ further adjusted for the quality of leadership, influence at work, work-privacy conflict, work stress (ERI), and phys. environmental and burdensome factors

^c^ further adjusted for self-rated health and language at home

^d^ reduction of effect size between model 0 and 3

Block-wise logistic regression modelling utilisation of rehabilitation in general was performed to investigate differences between these two groups, as shown in Table 4. This implicated a minor lower OR for foreign EMB compared to German EMB, though there were no significant group differences (fully adj. OR 0.91, 95% CI 0.57-1.46). After further adjusting the models, the probability of rehabilitation (AMEs) for foreign EMB (to -1.66%-points) decreased while holding the covariates at a constant value. However, in the final model 3, the probability declined again.

Secondary findings revealed that certain covariates had a significant association with the utilisation of rehabilitation. For all outcomes of rehabilitation, having poor health was associated with higher odds. Having a work-privacy conflict was associated with lower odds for the utilisation of outpatient rehabilitation while having low influence at work showed higher odds of using a rehabilitation in general. Further predictive factors with higher odds in several models were born in 1959, having medium work stress and having qualified or unskilled positions.

## Discussion

In the present study, we analysed the utilisation of medical rehabilitation and its subtypes (in- and outpatient) for subgroups of employees in relation to their migrant background. In the following, the main findings will be summarised. Subsequently, the results for the first research question comparing G1 und G2 EMB with non-EMB concerning their utilisation of general, inpatient and outpatient rehabilitation respectively will be discussed in chronological order. A discussion about the second research question, contrasting persons with foreign and German nationality with migrant employees will follow, as well as aspects about associated covariates to complete with the strengths and limitations of the present study.

Comparing G1 and G2 EMB with non-EMB, no significant group differences were found for the utilisation of general and inpatient rehabilitation. With respect to the utilisation of outpatient rehabilitation, however, G1 EMB had a 58% significantly lower chance than non-EMB when considering all explanatory covariates. The findings for G2 EMB were usually closer to those for non-EMB than to those for G1 EMB. Moreover, within EMB, foreign EMB showed a slightly lower but non-significant chance of using medical rehabilitation at all compared to German EMB.

To date, there are no other German studies investigating the utilisation of medical rehabilitation and its subtypes while differentiating migrant background, as detailed as in the presented study. Therefore, the following comparison to other German studies is only possible to a certain degree.

In other studies, where the differentiation in migrant background with large representative cohort data is not solely possible given the indicator of nationality but also other indicators, the results are as follows: Voigtländer et al. [20] analysed data from the Socio-Economic Panel (2002-2004) for Germany with the result that even after adjustment (e.g., for age, sex and socioeconomic status), the chance of using medical rehabilitation significantly decreased by 40% in persons with migrant background, compared to non-migrants, as well as for foreign nationals compared to Germans. Here, the authors defined migrant background slightly differently: more precisely as having a foreign nationality, being born abroad or with one parent born abroad, having double nationality or given German nationality after birth. Recent analyses by Brzoska with data from the Sociomedical Panel using differentiated indicators for migrant background independently of nationality (e.g. place of birth of the examined person and the parents, as well as the mother tongue) show a less frequent utilisation of rehabilitation among persons with migrant background, also after adjusting for covariates [25]. Finally, findings from a German telephone survey in 2002-2003 found that migrants who were born outside of Germany or who were born as non-German, had a lower utilisation rate of rehabilitation [48]. In contrast, for the first research question of our study, there were no differences found between G1 or G2 EMB compared to non-EMB for utilisation of rehabilitation in general. However, the distinction between G1 and G2 cannot be found in other studies on rehabilitation.

Concerning inpatient rehabilitation, the results of the lidA-study show that there are no significant group differences. However, we found 16% and 10% higher chances of using inpatient rehabilitation for G1 and G2 EMB, respectively, than for non-EMB. In the full model, the average marginal effects showed a larger difference in the probability of utilisation of inpatient rehabilitation between G1 EMB and non-EMB than between G2 EMB and non-EMB

Findings of higher utilisation for rehabilitation in EMB than in non-EMB have only been found for psychosomatic rehabilitation, including depression and somatisation, where foreign nationals, especially Turkish nationals, had a higher utilisation rate of psychosomatic rehabilitation than Germans [49–51]. However, these results are related to specific indications, and the data source only allows differentiation by nationality, making it not possible to compare the results.

Focusing on outpatient rehabilitation, G2 resembled non-EMB rather than G1 EMB, which might indicate the successful integration of the second-‘children’-generation of migrant employees in Germany. Most importantly, a significantly lower chance for G1 EMB to utilise this type of rehabilitation than non-EMB, even in the fully adjusted model, was detected.

The included covariates did not fully explain the differences in the model, while the difference in the AMEs between the null and the final model displayed an increase in the effect by 60%. Therefore, our findings indicate that these differences have to be attributable to factors other than sociodemographic, work- and non-work-related variables. These factors might be related to the rehabilitative care system and/or migrant-specific characteristics or understandings of health that go beyond differences in the considered patterns.

Thus far, research on possible barriers to the utilisation of medical rehabilitation for EMB has addressed access to barriers and barriers within medical rehabilitation. In particular, the lack of knowledge about the rehabilitation system and its possibilities are the main barriers to access, not only for EMB but also for general practitioners who recommend rehabilitation. At the same time, diverse treatment concepts that are sensitive to religion, culture and gender are missing. Discrimination and miscommunication, due to language barriers and illiteracy, are also barriers to the utilisation of rehabilitation by EMB [22–24].

No equivalent to the finding that G1 EMB have lower odds of using outpatient rehabilitation can be found in the existing studies. Only one review investigating inequalities in health care utilisation among migrants found that first-generation migrants have a lower utilisation of outpatient care, such as specialist consultations and physical therapy [52].

Most studies do not consider both, in- and outpatient medical rehabilitation separately. In Germany, comparable research has mainly focused on medical rehabilitation in general, summarising all types of rehabilitation. This may be due to lacking information about the different rehabilitation types in the data sets or to the lower number of cases not allowing for stratification. The latter is the result of a generally lower utilisation of outpatient medical rehabilitation services by adults compared to the utilisation of inpatient medical rehabilitation (ca. 80%) [4], which dominates in Germany [6]. Relevant characteristics of the rehabilitation systems differ substantially between countries. While in Germany, medical rehabilitation is dominated by inpatient rehabilitation, often far away from home, in other European countries the opposite is true: most rehabilitation services are outpatient services close to the persons’ homes. Such differences make it difficult to compare data on rehabilitation utilisation between various countries. Additionally, not only in Germany, but throughout Europe, an insufficient differentiation of persons with migrant background by migrant characteristics in routine data can be observed [53]. Positive exceptions are, e.g., the Netherlands and Norway, where information on nationality, country of birth and the parents’ country of birth (in the case of the Netherlands) are collected in process data [6, 54].

The results concerning the second research question comparing German and foreign nationals within the subsample of migrant employees are partly comparable to other studies. The findings are in line with previous results showing that foreign nationals utilise rehabilitation less often than Germans [19, 21, 55, 56]. Nevertheless, our analyses excluded persons without any migrant background from the group of German citizens, while other studies still include these persons, because of differentiating simply by nationality. Hence, the effect might be diluted and is clearly not the same as in our results, where EMB of foreign nationality had a lower but non-significantly different chance of rehabilitation compared to a German EMB. Separated analyses for inpatient and outpatient rehabilitation were not possible due to limited power. Even in the case of the utilisation of general rehabilitation (inpatient and outpatient combined), the number of events was fairly low in foreign EMB, which possibly contributed to our non-significant finding (Table 3).

Secondary findings revealed that certain covariates had a positive association with the utilisation of rehabilitation. Having poor health was associated in all models, while having a work-privacy conflict was only associated with outpatient rehabilitation. Further predictive factors were being born in 1959, having medium work stress, low influence at work and holding qualified or unskilled positions. All of them seem plausible, as they are congruent with reported findings so far [4–6, 19, 20, 23, 33].

Furthermore, this study has several strengths. First, the use of a national sample presents high representativeness for the population of socially insured employees of the considered two age cohorts [27]. Second, unlike other studies, the lidA cohort study has the ability to separate different migrant groups with several indicators and not only by nationality, so that recommendations for mapping migrant status can be followed [29]. The indicators used consisted of the participant’s country of birth, nationality and country of birth of each parent. Another strength of this study is the consideration of different confounding sociodemographic, work and individual variables that may disguise differences in the outcomes between the investigated groups. These should be considered in future studies, as it was found that EMB do not have the same levels of psychosocial resources as non-EMB [17], which are ultimately the important predictors of workability and rehabilitation. We still adjusted for language mainly spoken at home, as lacking knowledge of German was identified as a barrier to rehabilitation services and EMB might still have problems with the application process, although they were able to answer (part of) the interview questions. Furthermore, the usage and reporting of average marginal effects allows for direct comparisons between models of the same sample [46, 47]. Finally, the usage of multiple imputation by the fully conditional specified method presents another positive aspect of the analysis as the number of complete cases and statistical power could be increased, as well as bias due to missing values in certain of the variables reduced.

Despite these merits, there are some limitations of our study. The lidA cohort study uses two birth cohorts sampled within socially insured employees, which excludes civil servants, most self-employed persons as well as freelancers. As a result, the sample is limited regarding its representativeness of older employees in terms of age variety and occupational class. An additional restriction might have introduced a bias into participant selection, as the study was conducted in German and therefore EMB could be potentially excluded due to language problems. However, we assumed for these a certain knowledge of German when working in socially insured positions. Another possible weakness is the usage of the self-rated health status (SF-12) serviced after the potential rehabilitation, as health status prior to rehabilitation was unavailable to adjust as a covariate. Accordingly, the current health status was used as a proxy for the initial status, while assuming a similar health change for everyone who had used rehabilitation services so that the influence of the initial health status on rehabilitation utilisation would have been adequately adjusted for in the regression model. Last, the number of events (utilised rehabilitation) within the migrant groups included in the logistic regression analyses in relation to the number of events in the reference group was fairly low (e.g., 19 events in G2 EMB compared to 225 in non-EMB for outpatient rehabilitation), which should be considered when regarding the results.

## Conclusion

Our study has found that migrant employees of the first-generation utilise outpatient rehabilitation significantly less often than non-migrant employees. These findings are partly attributable to differences in sociodemographic, work- and non-work-related factors between these population groups. Other factors may play a role, possibly related to the rehabilitative care system, migrant-specific characteristics or understandings of health. Additionally, no significant differences between migrant employees of the first- or second-generation and non-migrant employees when comparing the utilisation of inpatient rehabilitation or any rehabilitation in Germany have been detected. The same was observed when analysing differences between German and foreign nationals within migrant employees. However, the migrant employees of the second-generation rather resemble the Germans than their parent generation (first-generation), which is an important fact regarding integration. Our distinct investigation contributes to the knowledge on the heterogeneity and different behaviours in the utilisation of health services such as medical rehabilitation. These results highlight the growing need to consider diversity sensitive services that are important for social-political decision makers to ensure equal opportunities and work participation. Further research should also consider the actual need for rehabilitation in employees with migrant background, as this could influence the utilisation patterns of rehabilitation and provide insights into their perceptions and coping with diseases.

## Supplementary information

**Additional file 1.** English version of self-developed questionnaire items used for analysis. The additional file contains the English translation of the self-developed questionnaire items used for analysis.

## Data Availability

The same datasets as analysed in the current study are available as a scientific use file at the research data centre of the German Federal Employment Agency at the Institute of Employment Research, which can be found here: https://fdz.iab.de/en/FDZ_Individual_Data/lidA.aspx and does not issue datasets with DOIs [28]. Additional information regarding the study as well as data documentation (data report and method report) are also available [27, 57, 58]

## Abbreviations

AME: Average marginal effect
CAPI: Computer Assisted Personal Interviews
EMB: Employees with migrant background
ERI: Effort-Reward-Imbalance
G1: First migrant generation
G2: Second migrant generation
lidA: ‘Leben in der Arbeit’
MD: Missing data
non-EMB: Employees without migrant background
non-PMB: Persons without migrant background
PMB: Persons with migrant background
