# Supplementary material for: Utilisation of rehabilitation services for non-migrant and migrant groups of higher working age in Germany – results of the lidA cohort study
Source: BMC Health Serv Res. 2020 Jan 10;20:31. doi: 10.1186/s12913-019-4845-z (PMC6954536; doi:10.1186/s12913-019-4845-z)
Supplement: Supplementary file 1 — Additional file 1. English version of self-developed questionnaire items used for analysis. The additional file contains the English translation of the self-developed questionnaire items used for analysis. [file 12913_2019_4845_MOESM1_ESM.pdf]

Additional file 1: English version of self-developed questionnaire items used for analysis

Questionnaire item	Translation of the question including response options
Sex	Sex of the respondent (not asked, registered only)<br>- male<br>- female
Year of birth	Please tell me when you were born? Please tell me the day, the month and the year.<br>__ day (two digits) __ month (two digits) ____ year (four digits)
Rehabilitation services	Have you utilised a medical rehabilitation since 2008?<br>- no<br>- yes, an inpatient treatment with accommodation in a rehabilitation hospital.<br>- yes, an outpatient treatment.
Migrant background	Were you born in Germany?<br>- yes<br>- no<br><br>In which country were you born? (open question)<br>- [country of birth]<br><br>What is your nationality?<br>- German<br>- Other, [nationality]<br>- dual nationality<br>- stateless<br><br>Our next questions refer to your parents. Was your father born in Germany?<br>- yes<br>- no<br>- father unknown, no step- or adoptive father etc.<br><br>Was your mother born in Germany?<br>- yes<br>- no<br>- mother unknown, no step- or adoptive mother etc.
Main language spoken at home	Which language do you normally speak at home?<br>- only German<br>- mostly German<br>- mostly another language<br>- only another language
